# Optimized classification of ^18^F-Florbetaben PET scans as positive and negative using an SUVR quantitative approach and comparison to visual assessment

**DOI:** 10.1016/j.nicl.2017.04.025

**Published:** 2017-05-13

**Authors:** Santiago Bullich, John Seibyl, Ana M. Catafau, Aleksandar Jovalekic, Norman Koglin, Henryk Barthel, Osama Sabri, Susan De Santi

**Affiliations:** a Piramal Imaging GmbH, Berlin, Germany; b Molecular Neuroimaging, New Haven, CT, USA; c Department of Nuclear Medicine, University Hospital Leipzig, Leipzig, Germany; d Piramal Pharma Inc., Boston, MA, USA

**Keywords:** Florbetaben, PET, SUVR, Quantification, Visual assessment

## Abstract

**Introduction:**

Standardized uptake value ratios (SUVRs) calculated from cerebral cortical areas can be used to categorize ^18^F-Florbetaben (FBB) PET scans by applying appropriate cutoffs. The objective of this work was first to generate FBB SUVR cutoffs using visual assessment (VA) as standard of truth (SoT) for a number of reference regions (RR) (cerebellar gray matter (GCER), whole cerebellum (WCER), pons (PONS), and subcortical white matter (SWM)). Secondly, to validate the FBB PET scan categorization performed by SUVR cutoffs against the categorization made by post-mortem histopathological confirmation of the Aβ presence. Finally, to evaluate the added value of SUVR cutoff categorization to VA.

**Methods:**

SUVR cutoffs were generated for each RR using FBB scans from 143 subjects who were visually assessed by 3 readers. SUVR cutoffs were validated in 78 end-of life subjects using VA from 8 independent blinded readers (3 expert readers and 5 non-expert readers) and histopathological confirmation of the presence of neuritic beta-amyloid plaques as SoT. Finally, the number of correctly or incorrectly classified scans according to pathology results using VA and SUVR cutoffs was compared.

**Results:**

Composite SUVR cutoffs generated were 1.43 (GCER), 0.96 (WCER), 0.78 (PONS) and 0.71 (SWM). Accuracy values were high and consistent across RR (range 83–94% for histopathology, and 85–94% for VA). SUVR cutoff performed similarly as VA but did not improve VA classification of FBB scans read either by expert readers or the majority read but provided higher accuracy than some non-expert readers.

**Conclusion:**

The accurate scan classification obtained in this study supports the use of VA as SoT to generate site-specific SUVR cutoffs. For an elderly end of life population, VA and SUVR cutoff categorization perform similarly in classifying FBB scans as Aβ-positive or Aβ-negative. These results emphasize the additional contribution that SUVR cutoff classification may have compared with VA performed by non-expert readers.

## Highlights

• SUVR cutoffs to classify Florbetaben PET scans as positive and negative were generated.
• SUVR cutoffs were validated against post-mortem histopathological confirmation.
• Added value of SUVR cutoff classification to visual assessment was evaluated.
• SUVR cutoff classification provided higher accuracy than some non-expert readers.
• Results emphasize the contribution that SUVR cutoffs may have to visual assessment.

## 1 Introduction

Visual assessment (VA) is typically used to classify ^18^F-Florbetaben (FBB) positron emission tomography (PET) scans as positive or negative for the presence of amyloid-beta (Aβ) in clinical practice and for subject screening in therapeutic clinical trials. However, a semi-quantitative approach using standardized uptake value ratios (SUVRs) calculated from selected cerebral cortical areas can also be used to categorize FBB PET scans by applying appropriate cutoffs (Barthel et al., 2011, Jennings et al., 2015, Ong et al., 2013, Sabri et al., 2015, Seibyl et al., 2016, Tuszynski et al., 2016, Villemagne et al., 2011). SUVR values of scans exceeding a certain threshold are classified as positive. Conversely, those scans with SUVR values below a threshold are classified as negative. Although this method is simple and operator independent, its current application is limited since it depends on a number of factors such as scan time after injection, image reconstruction and processing, partial volume correction, region-of-interest (ROI) delineation method, reference region (RR), and standard of truth used (SoT). Consequently, optimal SUVR cutoffs can differ between sites. SUVR cutoffs should be generated in-house or with a standardized method to ensure comparability between sites.

Site-specific SUVR cutoff generation is costly and time consuming since it involves image acquisition, quantification and selection of the threshold that provides optimal scans classification according to a given SoT. Ideally, histopathological confirmation of the Aβ presence in the brain should be used as SoT. However, this information is rarely available since it can only be obtained post-mortem. The use of clinical diagnoses as SoT offers another alternative. Nevertheless, clinical diagnosis as SoT may not be appropriate given its accuracy limitations. It has been reported that up to one-third of people clinically diagnosed with mild to moderate AD do not meet criteria for significant Aβ accumulation in the cerebral cortex (Beach et al., 2012, Monsell et al., 2015) while also some cognitively normal subjects may have elevated Aβ deposition in the brain. Given the difficulties of histopathology and clinical diagnoses, SUVR cutoff generation using VA as SoT is convenient as this information is readily available, correlates well with histopathological confirmation of the Aβ presence in the brain (Sabri et al., 2015) and may facilitate site-specific SUVR cutoffs. However, the performance of FBB SUVR cutoffs generated using VA as SoT has not been validated so far against histopathological confirmation of Aβ deposition in the brain.

Although a number of FBB SUVR cutoffs have been reported (Barthel et al., 2011, Jennings et al., 2015, Ong et al., 2013, Sabri et al., 2015, Seibyl et al., 2016, Villemagne et al., 2011), SUVR cutoff methodology still has some limitations. Firstly, the RR selection influences the reliable measurement of Aβ (Bullich et al., 2017) and although FBB SUVR cutoffs have been reported using cerebellar cortex as RR, little is known about their performance using other RRs (e.g. whole cerebellum, pons and subcortical white matter). Secondly, although high correlation has been reported to visual read (Seibyl et al., 2016), it is not known whether classification based on SUVR cutoffs can replace VA as screening tool in clinical trials or whether it can help to assess difficult scans in clinical practice. Indeed, the specific role of the cutoff value, whether for eligibility screening or optimized clinical diagnosis will determine the point selected on the receiver operating characteristic (ROC) curve.

The objective of this work was, thus, firstly to generate FBB SUVR cutoffs using VA as SoT for a number of RRs. Secondly, to validate the FBB PET scan categorization performed by SUVR cutoffs against the categorization made by post-mortem histopathological confirmation of the presence of Aβ. Finally, the added value of SUVR cutoff categorization to VA was evaluated.

## 2 Methods

### 2.1 Subjects, image acquisition and quantification

#### 2.1.1 Subjects

The study population consisted of 226 subjects who underwent FBB PET scans in previous multicenter clinical trials. These studies were conducted in accordance with the Declaration of Helsinki and after approval of the local ethics committees of the participating centers. The scans were grouped in two cohorts (Table 1). Cohort A comprised 143 subjects (69.5 ± 7.5 yrs (mean ± SD); n = 75 Alzheimer's disease (AD), n = 68 healthy volunteers) who underwent FBB PET scans and were visually assessed by 3 independent blinded readers (Barthel et al., 2011). Cohort B comprised 78 end-of-life subjects (80.1 ± 10.4 yrs; n = 56 (AD), n = 9 (non-demented volunteers), n = 13 (other dementias)) who underwent FBB PET imaging, had a visual assessment of their PET scans by eight independent blinded readers and who had a subsequent post-mortem neuropathological determination of Aβ load in the brain (Sabri et al., 2015).

**Table 1:** Subjects, SoT, validation references and performance measurement used for SUVR cutoff generation and validation.

	Subject	SoT	SUVR cutoff generation
SUVR cutoff generation (cohort A)	n = 143 (69.5 ± 7.5 yrs) • AD (n = 75) • Non-demented healthy volunteers (n = 68)	VA (majority read of 3 independent expert blinded readers)	ROC analysis


	Subject	Validation reference	SUVR cutoff performance measurement
SUVR cutoff validation (cohort B)	n = 78 end-of-life patients (80.1 ± 10.4 yrs) • AD (n = 56) • Other dementia (n = 13) • Non-demented healthy volunteers (n = 9)	Histopathology for neuritic plaques • Stains: BSS/IHC^a^ • Consensus panel (n = 3 expert pathologists) VA (majority read from eight blinded independent readers)	Sensitivity, specificity and accuracy. Percent agreement between SUVR cutoffs and VA

a BSS: Bielschowsky silver stain; IHC: immunohistochemistry.

#### 2.1.2 Image acquisition and reconstruction

A 3D Hoffmann brain phantom was acquired prior to subject enrollment in order to establish a standardized acquisition and reconstruction method for ensuring comparability of quantitative PET between sites. All subjects underwent a 20 min PET scan (4 × 5 min dynamic frames) starting at 90 min after intravenous injection of 300 MBq ± 20% of FBB followed by a 10 mL saline flush. PET scans were reconstructed using Ordered Subsets Expectation Maximization (OSEM) algorithm using 4 iterations and 16 subsets (zoom = 2) or comparable reconstruction as guided by the phantom. Corrections were applied for attenuation, scatter, randoms and dead time. Three-dimensional volumetric T1-weighted brain magnetic resonance imaging (MRI) data (e.g. magnetization prepared rapid gradient echo (MPRAGE) or spoiled gradient recalled (SPGR) sequences) was also collected.

#### 2.1.3 Image analysis

Image processing was performed as previously described by Barthel et al. (2011). The average activity was calculated in the ROIs placed on the cerebellar gray matter (GCER), cerebellar white matter, subcortical white matter (SWM), pons and cerebral cortical regions (frontal, occipital, parietal, lateral temporal and posterior and anterior cingulate cortex regions). Whole cerebellum (WCER) activity was generated by averaging the activity in the cerebellar gray matter and cerebellar white matter. SUVR was calculated as the ratio of the activity in the cerebral cortical regions and the activity of four different RRs (GCER, WCER, PONS and SWM). A composite SUVR was calculated for each RR by averaging the SUVR of 6 cortical regions (frontal, occipital, parietal, lateral temporal and posterior and anterior cingulate cortex regions) (Rowe et al., 2008).

### 2.2 Visual assessment

All blinded readers followed the same reading methodology as previously described by Seibyl et al. (2016). Tracer uptake was assessed in four cortical regions (lateral temporal cortex, frontal cortex, parietal cortex and posterior cingulate cortex/precuneus) according to the regional cortical tracer uptake (RCTU) system (Supplementary Table 1). Subsequently, the global uptake in the brain was assessed according to the brain amyloid plaque load (BAPL) system (Supplementary Table 2). The final result of the VA was based on the majority read (i.e. agreement of the majority of readers). Cohort A and B were read by 3 independent blinded readers with previous extensive experience reading FBB scans trained in-person. Additionally, cohort B was read by 5 naïve independent blinded readers without previous experience reading FBB scans that were trained using the FBB electronic training program prior to the reading session. In cohort B, where 8 readers assessed the PET scans, majority reads could be established in all the cases and none of the scans presented a draw (4 positive/4 negative).

### 2.3 Post-mortem histopathology

Brain samples from subjects in cohort B who died during the study were used to obtain histopathological confirmation of Aβ presence in the brain, as previously described in Sabri et al. (2015). From all the brain regions analyzed by post-mortem histopathology only those that were also visually assessed (frontal cortex and posterior cingulate cortex) were considered in the study. Neuritic/cored Aβ was classified as present in a given brain region when scored as “moderate” or “frequent” either by BSS or IHC. Neuritic/cored Aβ was classified as absent in a given brain region when scored as “none” or “sparse” by both BSS and IHC.

### 2.4 SUVR cutoff generation

SUVR cutoffs were generated with a ROC analysis to ascertain the optimal threshold for the sensitivity and specificity calculation using data from cohort A. The SUVR that provided the highest Youden's index (sensitivity + specificity − 1) was selected. SUVR cutoffs were generated for the composite and for several individual cortical regions (frontal cortex, lateral temporal cortex, parietal cortex and posterior cingulate cortex) using four RRs (GCER, WCER, PONS and SWM). To generate regional SUVR cutoffs, SoT was based on the classification performed according to the RCTU score system (Aβ absent (RCTU = 1), Aβ present (RCTU = 2 or 3)). To generate composite SUVR cutoffs, SoT was based on the classification performed according the BAPL score system (absent (BAPL = 1) and present (BAPL = 2 or 3)).

### 2.5 SUVR cutoff validation

The performance of SUVR cutoffs to categorize FBB PET scans was validated in cohort B against post-mortem histopathological determination of the Aβ deposition. Firstly, sensitivity, specificity and accuracy of SUVR cutoff categorization against histopathological determination of Aβ deposition were calculated. Secondly, the percentage of agreement between VA and SUVR cutoffs for different RR was obtained. Finally, the added value of SUVR cutoff categorization to visual read was evaluated. The performance of VA and SUVR cutoffs to categorize FBB PET scans against histopathology confirmation was compared.

### 2.6 Statistical analysis

The performance of SUVR cutoffs to classify FBB PET scans was assessed by means of the sensitivity, specificity and accuracy and their 95% confidence intervals (CIs). Percentage of agreement and the 95% CIs was used to compare SUVR cutoffs, VA and histopathological confirmation of Aβ deposition. CIs were obtained by using the Clopper and Pearson procedure (Clopper and Pearson, 1934). Statistical differences across target and RRs were analyzed by using the chi-squared test between pairs and corrected for multiple testing using Bonferroni-Holm method (Holm, 1979). A p-value lower than 0.05 was considered significant. Statistical analysis was performed using R.

## 3 Results

### 3.1 SUVR cutoff generation

**Fig. 1:**
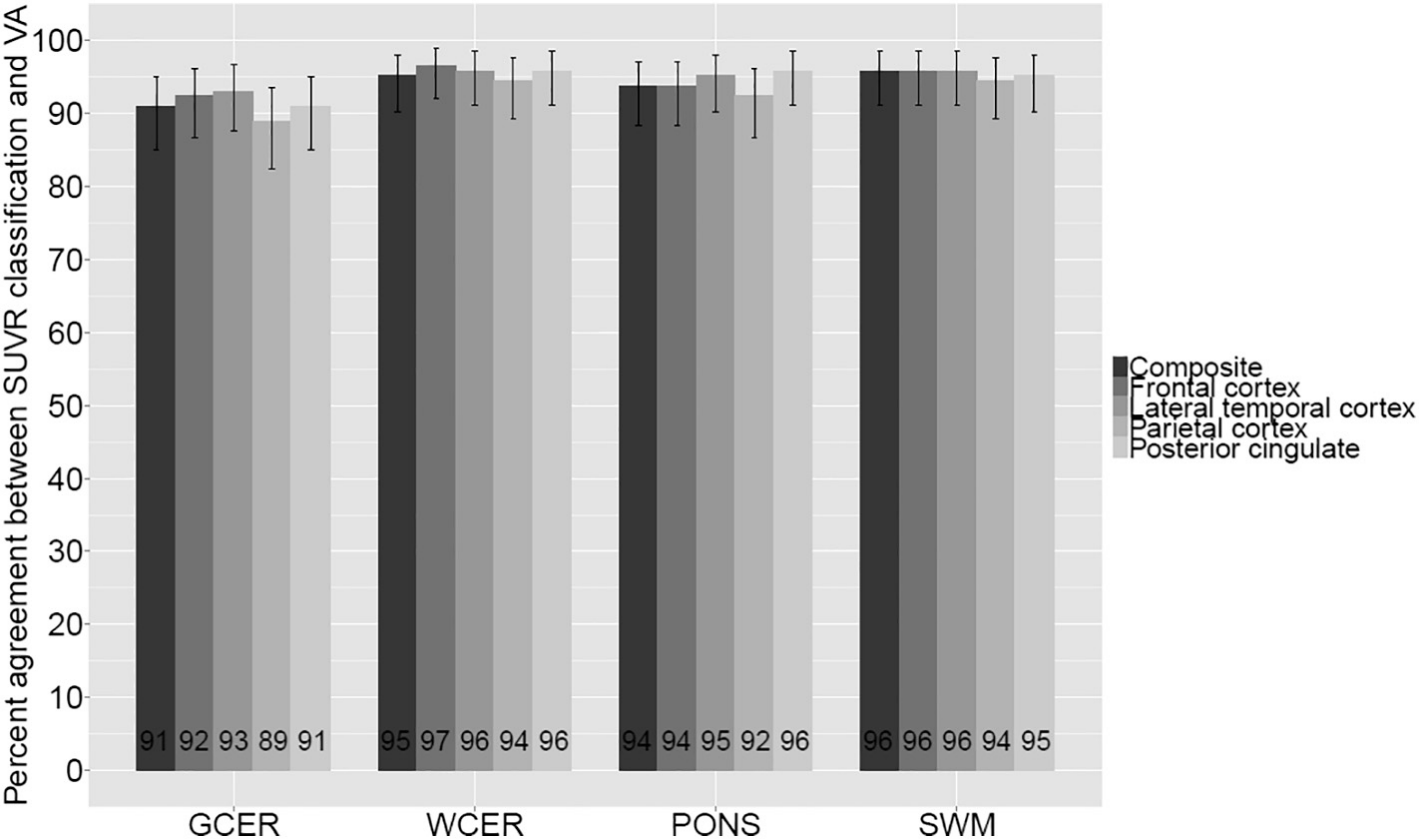
Percentage of agreement (and 95% confidence interval) between different SUVR cutoff classifications and VA in cohort A.

Composite (SUVR_GCER_ = 1.43, SUVR_WCER_ = 0.96, SUVR_PONS_ = 0.78, and SUVR_SWM_ = 0.71) and regional SUVR cutoffs were generated using VA as SoT and different RRs (Table 2). The percentage of agreement between scan classification based on SUVR cutoffs and VA was high for all RR (range 89–97%) (Fig. 1). No significant differences were found across RRs for composite or any cortical region.

**Table 2:** SUVR cutoffs generated for different cortical and reference regions and area under the ROC (in parentheses).

	Reference region			
	GCER	WCER	PONS	SWM
Composite^a^	1.43 (0.94)	0.96 (0.98)	0.78 (0.96)	0.71 (0.97)
Frontal cortex	1.43 (0.93)	0.93 (0.97)	0.76 (0.95)	0.69 (0.94)
Lateral temporal cortex	1.43 (0.95)	0.93 (0.97)	0.77 (0.97)	0.66 (0.96)
Parietal cortex	1.35 (0.95)	0.98 (0.99)	0.71 (0.94)	0.68 (0.83)
Posterior cingulate cortex	1.63 (0.93)	1.10 (0.99)	0.88 (0.97)	0.80 (0.99)

a Composite region = mean SUVR of 6 cortical regions (frontal, occipital, parietal, lateral temporal, anterior and posterior cingulate cortex regions).

### 3.2 SUVR cutoff validation

**Fig. 2:**
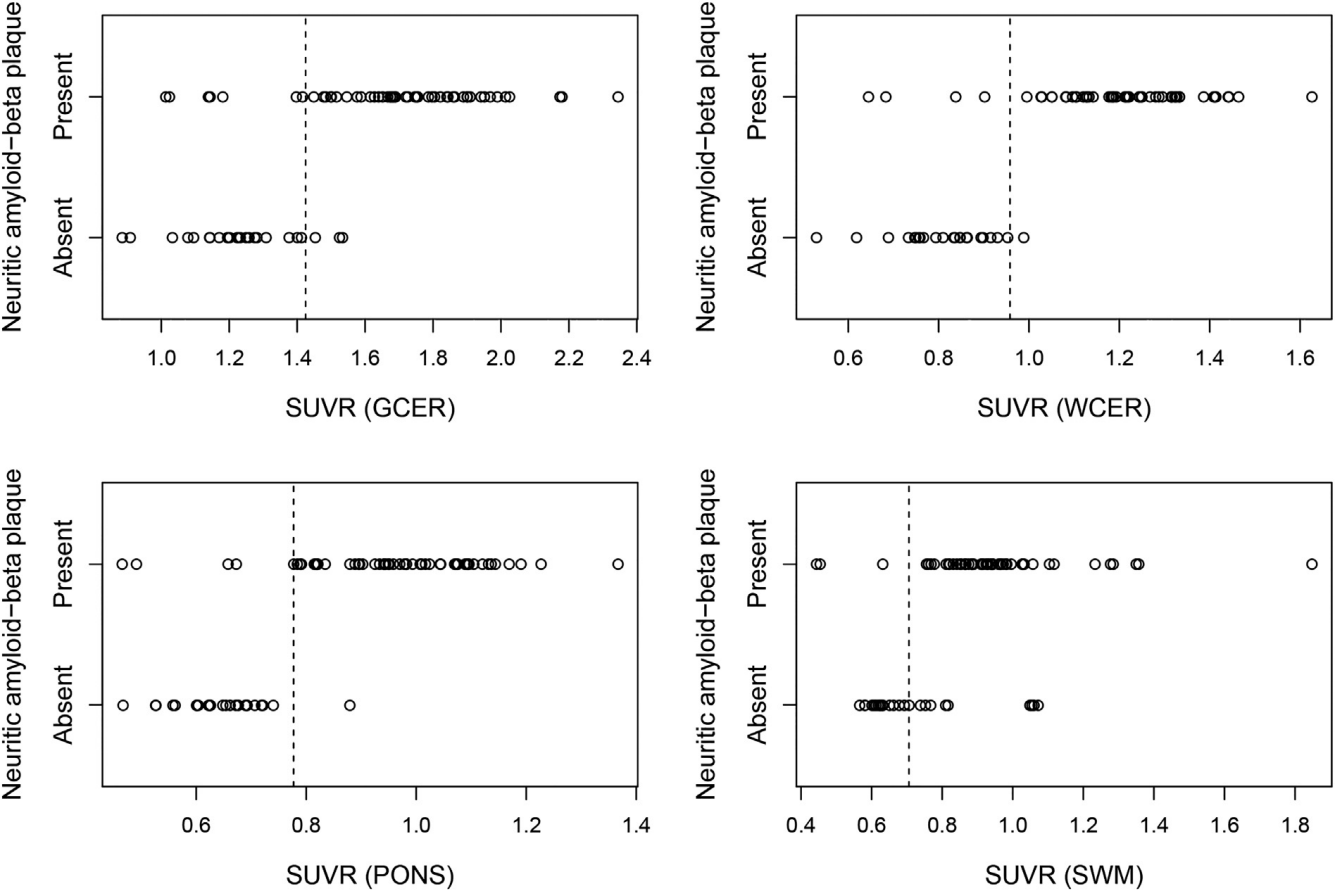
SUVR values versus the histopathological determination of neuritic Aβ in cohort B. Black dashed lines represent the SUVR cutoffs generated in cohort A using VA as SoT.

Performance of generated SUVR cutoffs was validated in cohort B against histopathological determination of the presence of neuritic Aβ (Fig. 2). For the composite SUVR, both WCER and PONS provided same high values of sensitivity (92%), specificity (96%) and accuracy (94%) but not significantly different from the GCER (sensitivity = 87%, specificity = 88%, accuracy = 87%) (Table 3). In contrast, SWM showed statistically significant lower specificity (60%) than WCER (p = 0.04) and PONS (p = 0.04). Regional SUVR cutoffs (frontal cortex and posterior cingulate cortex) provided high sensitivity but did not improve the overall classification performance of composite.

**Table 3:** Results of SUVR cutoff categorization (and 95% confidence intervals) of 18F-Florbetaben PET scans compared to histopathological determination of Aβ in the brain.

Target region	Reference region	Sensitivity	Specificity	Accuracy
Composite^a^	GCER	87 (75–95)	88 (69–97)	87 (78–94)
	WCER	92 (82–98)	96 (80–100)	94 (86–98)
	PONS	92 (82–98)	96 (80–100)	94 (86–98)
	SWM	94 (84–99)	60 (39–79)^b^, ^c^	83 (73–91)
Frontal cortex	GCER	89 (75–97)	72 (47–90)	84 (72–92)
	WCER	97 (86–100)	72 (47–90)	89 (78–96)
	PONS	92 (79–98)	72 (47–90)	86 (74–94)
	SWM	95 (82–99)	53 (28–77)	82 (69–91)
Posterior cingulate cortex	GCER	86 (68–96)	58 (37–77)	73 (59–84)
	WCER	93 (77–99)	58 (37–77)	76 (63–87)
	PONS	97 (83–100)	62 (41–80)	80 (67–90)
	SWM	100 (88–100)	24 (9–45)	65 (51–77)

a Composite region = mean SUVR of 6 cortical regions (frontal, occipital, parietal, lateral temporal and posterior and anterior cingulate cortex regions).

b Statistically significant differences with respect to WCER.

c Statistically significant differences with respect to PONS.

### 3.3 Comparison of visual and quantitative assessments

**Fig. 3:**
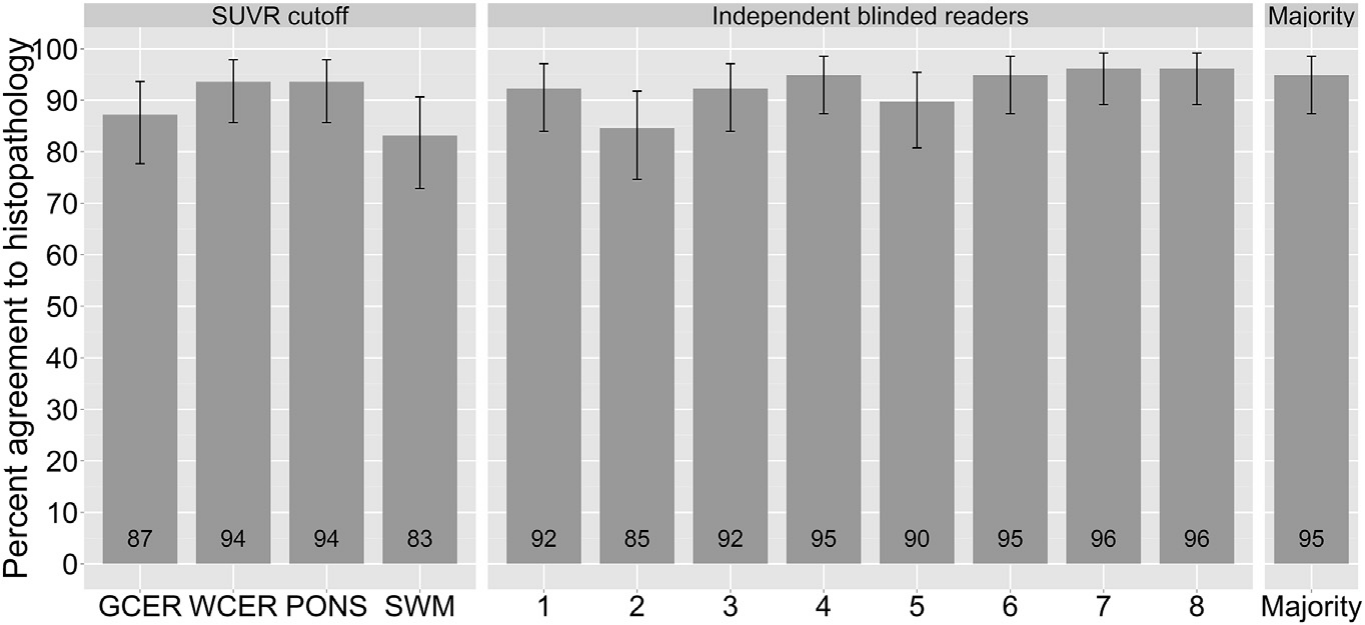
Percent agreement (and 95% confidence intervals) of histopathological confirmation of Aβ in the brain to SUVR cutoff categorization and visual assessment (independent blinded readers and majority read). Independent blinded readers 1–5 were naïve readers, trained via an electronic reader training program. Independent blinded readers 6–8 were expert readers, trained via in-person reader training program.

High percentage of agreement was obtained in the scan classification between composite SUVR cutoffs and VA majority read in cohort B (87% (GCER), 91% (WCER), 94% (PONS) and 85% (SWM)). In the subsample where all the 8 blinded readers had consensus (n = 60), the concordance was even higher (92% (GCER), 95% (WCER), 97% (PONS) and 90% (SWM)). SUVR cutoff and VA provided high agreement to histopathological determination of Aβ deposition in the brain (Fig. 3). SUVR cutoff classification using WCER and PONS showed higher agreement to histopathology (94%) than VA in 4 out of 5 non-expert blinded readers (85–95%) (Fig. 3). However, SUVR cutoff did not improve categorization compared to VA majority read (95%) or expert readers (95–96%). WCER and PONS were the RRs that provided a classification closest to the VA majority read. SUVR cutoff using SWM as RR performed significantly worse (83%) than VA majority read (95%) (χ^2^ = 5.36, p = 0.02).

## 4 Discussion

Optimal SUVR cutoffs may be distinct from site to site due to the use of different equipment, image acquisition, and processing. In this study, SUVR cutoffs were generated using VA as SoT and their performance to classify FBB scans was assessed against histopathological determination of Aβ in the brain. The accurate scan classification obtained supports the use of VA as SoT, and therefore allows to overcome the difficulties arising from other SoTs (e.g. histopathology or clinical diagnosis) and facilitates in-house SUVR cutoff generation. The composite SUVR cutoffs generated in this study (SUVR_GCER_ = 1.43) are in concordance with those previously reported (1.39 (Barthel et al., 2011), 1.478 (Sabri et al., 2015), 1.4 (Villemagne et al., 2011), 1.45 (Ong et al., 2013)) which emphasizes the robustness of FBB SUVR cutoffs. However, to the best of our knowledge, this manuscript analyses and validates for the first time the performance of FBB SUVR categorization in a sample different than the sample used to generate the SUVR cutoff. Additionally, the high agreement between FBB SUVR categorization and histopathology (92% sensitivity and 96% specificity using WCER as RR) and visual interpretation (percent agreement = 94% (WCER)) is in line with the results reported for other amyloid radioligands (Clark et al., 2012, Thurfjell et al., 2014). Thurfjell et al. reported for flutemetamol a good agreement between automated PET-only quantification and histopathologic classification of neuritic plaque density (91% sensitivity and 88% specificity using pons as RR) and visual read results (percent agreement = 97.1–99.4%) (Thurfjell et al., 2014). Similar results were reported for florbetapir where the use of a semi-quantitative approach resulted in an accuracy of 97% in relation to autopsy (Clark et al., 2012).

The present study assessed the SUVR cutoff classification performance for a number of RRs. A RR in amyloid PET should have the same non-displaceable activity (free + nonspecific binding) and similar blood flow characteristics as the target region, and should be amyloid-free (Schmidt et al., 2015). These requirements are fulfilled by cerebellar gray matter, except in patients with advanced stage of AD and in some types of familial AD in which cerebellar Aβ aggregates might occur (Knight et al., 2011, Thal et al., 2002). However, the cerebellar gray matter is likely to be devoid of Aβ in the clinical intended population for brain Aβ imaging. Additionally, the effect of cerebellar plaques in cortical FBB SUVRs appears to be negligible even in advanced stages of AD with high cortical Aβ load (Catafau et al., 2016). For this reason, GCER is commonly used as RR for relative FBB uptake quantification. However, no statistically significant differences between GCER, WCER and PONS were found in this study suggesting the robustness of all these three RRs for SUVR cutoff classification. In contrast, SWM provided lower classification performance than WCER and PONS when compared to histopathology confirmation. Relative FBB uptake quantification using the SWM is likely to be affected by atrophy and vascular lesions which are less frequently found in cerebellum and pons. Additionally, white matter could play a specific role in amyloid compound uptake. For example, white matter histogram analysis revealed significant differences between AD and healthy subjects using florbetapir PET indicating that binding in white matter conveys subtle information not detectable using the SUVR approach (Nemmi et al., 2014). However, this cohort of elderly end-of-life patients used to obtain histopathology data is not the intended-use population undergoing FBB assessment of brain Aβ burden and this may have affected the results. Noticeably, the agreement between SWM SUVR cutoffs and VA in a more relevant clinical sample (cohort A) was equivalent to the other three RRs. This good agreement of RR SWM in cohort A can be explained as consequence of the VA method used for FBB, which is likewise focused on the comparison of tracer accumulation in gray and white matter. It must be taken into account, however, that the good performance of GCER, WCER and PONS in this study refers only to their scan classification performance. However, RR recommendations should also take other aspects such as biological meaningfulness, test-retest variability, correlation with histopathology and capacity to detect subtle longitudinal changes into account. Some of these aspects have been assessed previously (Barthel et al., 2015, Bullich et al., 2017, Villemagne et al., 2015). Barthel et al. reported high correlation between SUVR and histopathologic confirmation of the Aβ status using GCER, WCER and PONS (Barthel et al., 2015), while Bullich et al. reported better performance of cerebellar RRs (GCER and WCER) than PONS and SWM for detecting subtle longitudinal changes (Bullich et al., 2017). Finally, Villemagne et al. reported the highest SUV stability across time, across clinical conditions and across cerebral Aβ status for FBB when using CGM as RR (Villemagne et al., 2015).

**Fig. 4:**
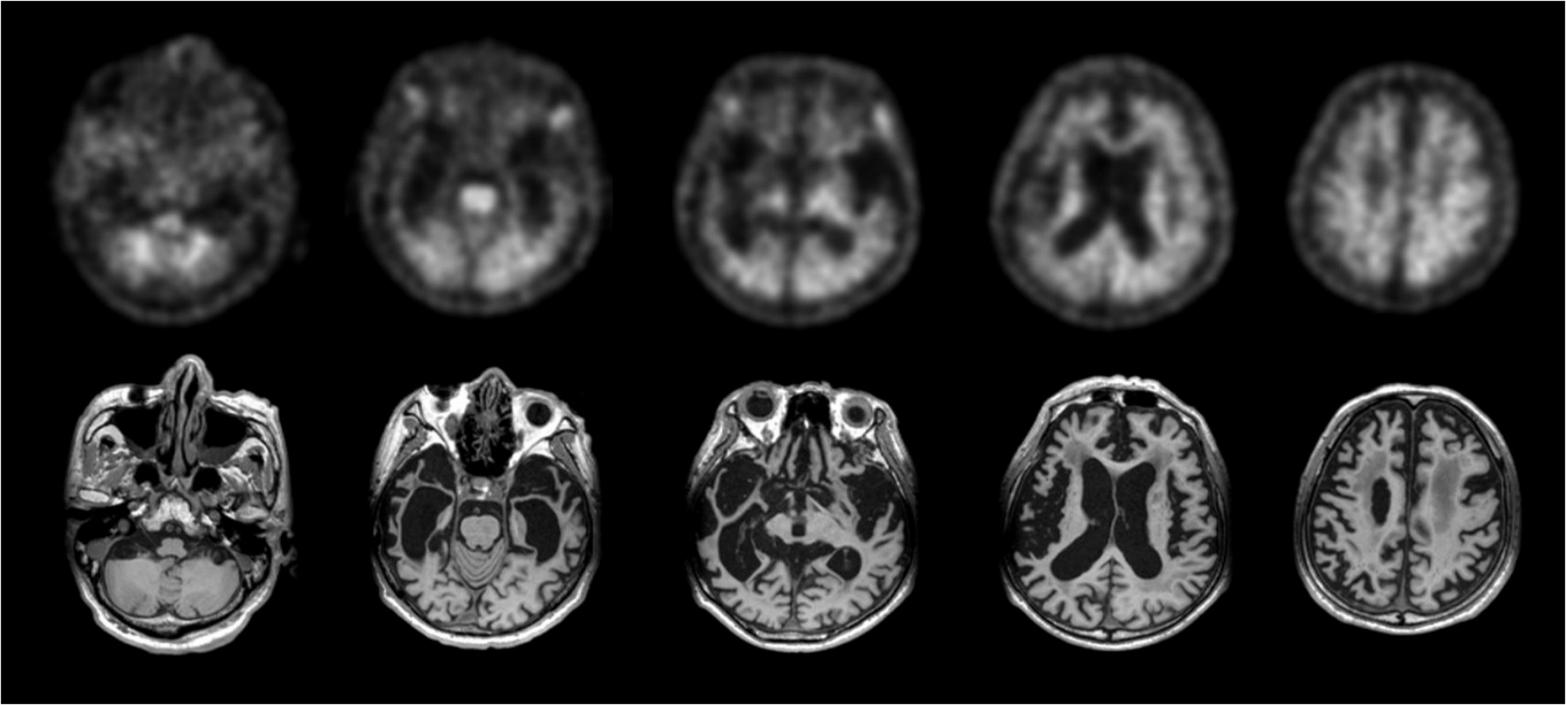
False-negative scan characterized with marked atrophy (subject #12, Table 4). Quantitative assessment of the scan using GCER and WCER was negative (SUVR_GCER_ = 1.14, SUVR_WCER_ = 0.90 (composite)) while visual assessment majority read and histopathological confirmation was positive.

**Fig. 5:**
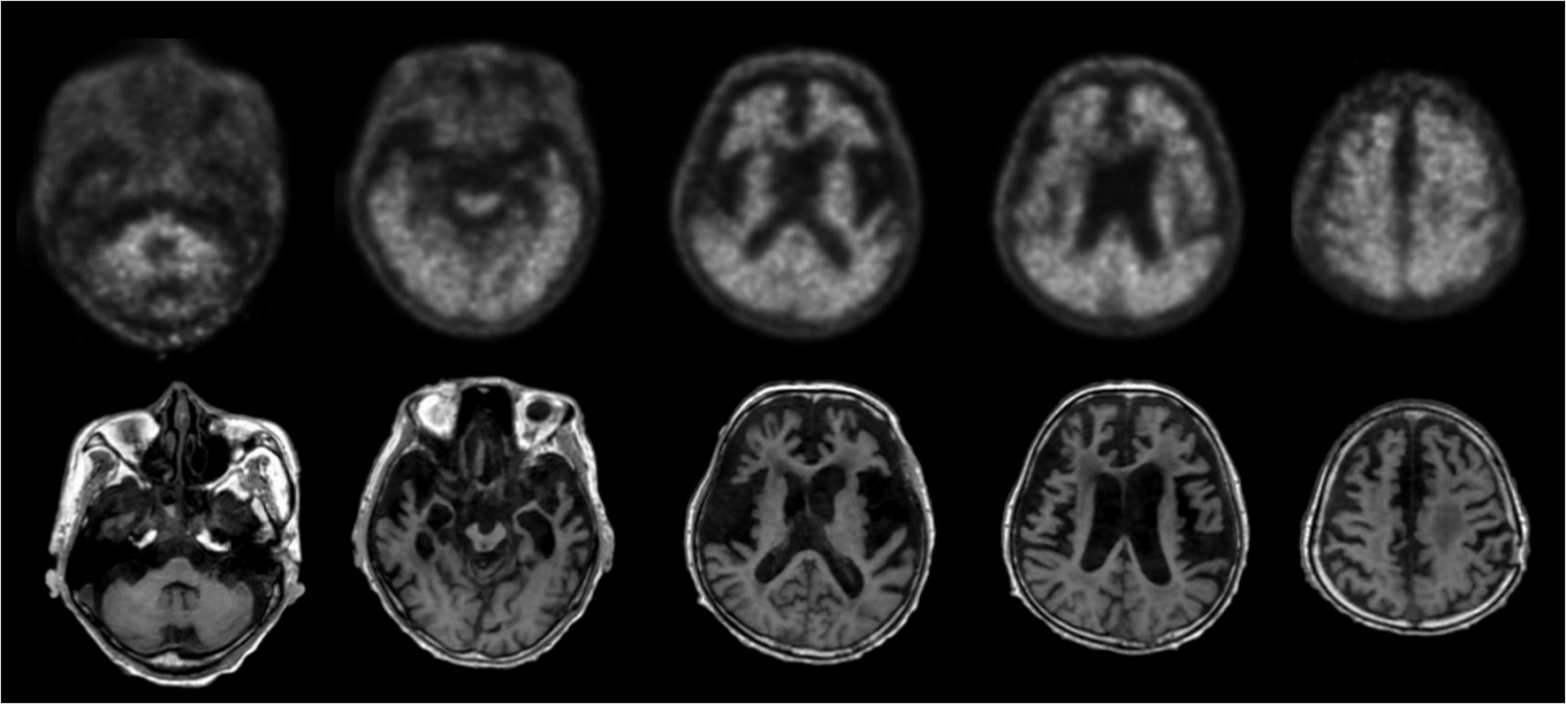
False-positive scans when using visual assessment and SUVR with GCER, WCER, PONS and SWM as RR (SUVR_GCER_ = 1.53, SUVR_WCER_ = 0.99, SUVR_PONS_ = 0.88, SUVR_SWM_ = 1.05 (composite)) (subject 16, Table 4). Histopathological confirmation was negative for the presence of neuritic Aβ but showing frequent diffuse Aβ.

A possible limitation of this study is the use of cohort A without proven diagnosis for SUVR cutoff generation instead of cohort B with histopathologic confirmation of Aβ status used only for validation. The reason of this design was to validate the SUVR cutoff generation in the clinical setting where histopathology or proven diagnosis is not available. Nevertheless, the SUVR cutoff obtained from cohort A (SUVR = 1.43 (composite)) is very similar to the optimal SUVR cutoff obtained from cohort B (SUVR = 1.47 (composite)) indicating the robustness of the SUVR cutoffs generated using VA. Moreover, the elderly end-of-life population that was required to obtain histopathological confirmation of Aβ status in the brain (cohort B) is different from the clinically intended population for Aβ PET scanning, which will likely be devoid of the brain structural abnormalities commonly found in elderly end-of life subjects. Despite the challenging quantification of some scans, the performance of SUVR cutoff categorization to classify FBB scans for the presence of Aβ was high for all the RRs. Additionally, high concordance was achieved between visual majority read and the SUVR cutoff even though the requirements applied to VA were demanding (i.e. scans only in the axial orientation, with no structural CT/MRI scans). SUVR cutoff, independent of the RR used, did not improve VA classification of FBB scans read either by the three expert readers or visual majority read of eight readers. Nevertheless, SUVR cutoff categorization provided higher accuracy in 4 out of 5 non-expert readers. These results emphasize the robustness of VA performed by expert readers and the additional contribution that optimized relative FBB uptake quantification may have for the detection of neuritic Aβ plaques by non-expert readers. An overview of the cases where SUVR cutoff failed to classify the scans is provided in Table 4. The errors in scan classification performed by SUVR cutoff categorization can be attributed to several reasons such us structural abnormalities (e.g. marked atrophy), challenging cases where readers did not reach consensus assessment (18 out of 78 scans (23.1%)) or borderline cases indicating a low amount of neuritic Aβ. The FBB PET and MR images of one such case in which marked atrophy was present is shown in Fig. 4. The brain images of another challenging case in which the PET scan was classified as negative despite the presence of substantial amount of diffuse Aβ are shown in Fig. 5. VA majority read was more accurate than SUVR cutoff categorization in those challenging cases. Further investigation is needed to substantiate whether more sophisticated quantitative methods (e.g. SUVR calculations using partial volume effect correction (Rullmann et al., 2016) or machine learning algorithms (Cattell et al., 2016)) can further assist VA categorization of such cases. Finally, this study does not address how a clinical reader would use the quantitative information in addition to, or adjunct to visual interpretation, nor the impact of combining visual and quantitative assessments on the overall scan assessment. Such algorithms require further research and validation, but may represent the future of clinical practice.

**Table 4:** Subjects incorrectly classified by at least one semi-quantitative FBB PET analysis compared to histopathology as SoT.

Case	Clinical diagnoses	Age (yrs)	SUVR_GCER_	SUVR_WCER_	SUVR_PONS_	SUVR_SWM_	Neuritic Aβ	VA	Assesment SUVR_GCER_	Assessment SUVR_WCER_	Assessment SUVR_PONS_	Assessment SUVR_SWM_	Assessment VA	Consensus^a^	Atrophy	Comments
1	AD patient	82	1.01	0.64	0.49	0.44	Present	Positive	FN	FN	FN	FN	TP	0/8	Marked	
2	AD patient	90	1.02	0.68	0.47	0.45	Present	Positive	FN	FN	FN	FN	TP	0/8	Marked	
3	AD patient	68	1.20	0.85	0.63	0.81	Absent	Negative	TN	TN	TN	FP	TN	6/2		Patient movement
4	AD patient	62	1.14	0.85	0.71	0.74	Absent	Negative	TN	TN	TN	FP	TN	8/0	Marked	
5	AD patient	98	1.67	1.13	0.81	0.76	Present	Negative	TP	TP	TP	TP	FN	6/2		Poor image quality
6	Other dementia	81	1.41	0.90	0.69	0.75	Absent	Negative	TN	TN	TN	FP	TN	7/1		Poor image quality
7	AD patient	58	0.89	0.62	0.47	0.82	Absent	Negative	TN	TN	TN	FP	TN	8/0	Marked	
8	non-demented	97	1.40	1.00	0.67	0.63	Present	Negative	FN	TP	FN	FN	FN	5/3		
9	DLB patient	73	1.45	0.89	0.62	0.58	Absent	Negative	FP	TN	TN	TN	TN	6/2		
10	Other dementia	83	1.52	0.86	0.60	0.68	Absent	Negative	FP	TN	TN	TN	TN	7/1	Marked	
11	AD patient	82	1.42	1.10	1.09	1.23	Present	Positive	FN	TP	TP	TP	TP	0/8	Marked	
12	Other dementia	70	1.14	0.90	0.79	0.88	Present	Positive	FN	FN	TP	TP	TP	0/8		
13	AD patient	84	1.03	0.75	0.67	1.05	Absent	Negative	TN	TN	TN	FP	TN	8/0	Marked	
14	AD patient	92	1.18	0.84	0.66	NA	Present	Positive	FN	FN	FN	NA	TP	1/7	Marked	
15	AD patient	72	1.14	1.08	0.78	1.85	Present	Positive	FN	TP	TP	TP	TP	0/8	Marked	
16	AD patient	86	1.53	0.99	0.88	1.05	Absent	Positive	FP	FP	FP	FP	FP	0/8	Marked	
17	Other dementia	75	0.91	0.76	0.72	1.07	Absent	Negative	TN	TN	TN	FP	TN	6/2	Marked	
18	AD patient	81	1.17	0.92	0.69	0.77	Absent	Positive	TN	TN	TN	FP	FP	3/5		
19	Non-demented	95	1.14	0.86	0.68	1.06	Absent	Negative	TN	TN	TN	FP	TN	8/0		
20	AD patient	76	1.28	0.95	0.74	0.71	Absent	Negative	TN	TN	TN	FP	TN	6/2		Poor image quality

SUVR: standardized uptake value ratio; Neuritic Aβ: histopathological determination of neuritic amyloid-β with BSS/IHC; GCER: cerebellar gray matter, WCER: whole cerebellum, SWM: subcortical white matter; VA: visual assessment, TP: true-positive; TN: true-negative; FP: false-positive; FN: false-negative.

a Number of independent blinded readers that assessed the scan as negative and positive (negative/positive).

## 5 Conclusion

The accurate scan classification obtained in this study supports the use of VA as SoT to generate site-specific SUVR cutoffs. For an elderly end of life population, VA and SUVR cutoff categorization perform similarly in classifying FBB scans as Aβ-positive or -negative. However, SUVR cutoff, independent of the RR used, did not improve VA classification of FBB scans read either by expert readers or majority read but provided higher accuracy than some non-expert readers. These results emphasize the additional contribution that optimized relative FBB uptake quantification using SUVR cutoffs may have to VA performed by non-expert readers.

## Funding

The trial was funded by Bayer Pharma AG, Berlin (Germany), and Piramal Imaging S.A., Matran (Switzerland).

## Conflict of interests

Santiago Bullich, Aleksandar Jovalekic and Norman Koglin are Piramal Imaging GmbH employee. Ana M. Catafau was Piramal Imaging GmbH employee until January 2016. Susan De Santi is employee of Piramal Pharma Inc. Henryk Barthel and Osama Sabri received research support, consultant honoraria, and travel expenses from Piramal Imaging GmbH. John Seibyl holds equity in Molecular Neuroimaging, a division of inviCRO.

## Ethical approval

All procedures performed in studies involving human participants were in accordance with the ethical standards of the institutional and/or national research committee and with the 1964 Helsinki declaration and its later amendments or comparable ethical standards.

## Informed consent

Informed consent was obtained from all individual participants included in the study.

## Appendix A Supplementary data

Supplementary tables Image 1
