# Supplementary material for: Optimized classification of 18F-Florbetaben PET scans as positive and negative using an SUVR quantitative approach and comparison to visual assessment
Source: Neuroimage Clin. 2017 May 13;15:325–32. doi: 10.1016/j.nicl.2017.04.025 (PMC5440277; doi:10.1016/j.nicl.2017.04.025)
Supplement: Supplementary file 1 — Supplementary tables [file mmc1.docx]

**Supplementary table 1.** Regional cortical tracer uptake (RCTU) score system used for visual assessment

| Regional assessment | Rule for assessment | | RCTU score |
| --- | --- | --- | --- |
| Normal/Negative  (no tracer uptake) | Tracer uptake (i.e., signal intensity) in grey matter in the region is lower than in white matter. | | 1 |
| Abnormal/Positive  (tracer uptake) | Tracer uptake equal to or higher than that present in white matter: extending beyond the white matter rim to the outer cortical margin involving the majority of the slices within the respective region.  The extension of the tracer uptake on the axial view of the region allows discrimination between moderate and pronounced uptake. | Moderate uptake: affecting a portion of the region | 2 |
|  |  | Pronounced uptake: affecting the whole region | 3 |

**Supplementary table 2.**  Brain amyloid plaque load (BAPL) score system for visual assessment.

| Scan assessment | BAPL Score | | Rule for assessment |
| --- | --- | --- | --- |
| Normal/Negative | 1 | Scan without Aβ deposition | RCTU score 1 in each of the 4 brain regions visually assessed. |
| Abnormal/Positive | 2 | Scan with moderate Aβ deposition | RCTU score 2 in any or all of the 4 brain regions visually assessed and no RCTU score 3 in these regions. |
|  | 3 | Scan with pronounced Aβ deposition | RCTU score 3 in at least one of the 4 brain regions visually assessed. |
